# Postretrieval Microinjection of Baclofen Into the Agranular Insular Cortex Inhibits Morphine-Induced CPP by Disrupting Reconsolidation

**DOI:** 10.3389/fphar.2020.00743

**Published:** 2020-05-19

**Authors:** Kuisheng Sun, Qingchun Mu, Haigang Chang, Chun Zhang, Yehua Wang, Shikuo Rong, Shenhai Liu, Di Zuo, Zhenquan He, Ding Wan, Hua Yang, Feng Wang, Tao Sun

**Affiliations:** ^1^ Ningxia Key Laboratory of Cerebrocranial Disease, Incubation Base of National Key Laboratory, Ningxia Medical University, Yinchuan, China; ^2^ Department of Neurosurgery, General Hospital of Ningxia Medical University, Yinchuan, China; ^3^ Department of Neurosurgery, The People's Hospital of Gaozhou, Gaozhou, China; ^4^ Department of Critical Care Medicine, The People's Hospital of Ningxia Hui Autonomous Region, Yinchuan, China

**Keywords:** morphine addiction, reconsolidation, conditioned place preference, agranular insular cortex, baclofen

## Abstract

Environmental cues associated with drug abuse are powerful mediators of drug craving and relapse in substance-abuse disorders. Consequently, attenuating the strength of cue-drug memories could reduce the number of factors that cause drug craving and relapse. Interestingly, impairing cue-drug memory reconsolidation is a generally accepted strategy aimed at reducing the intensity of cues that trigger drug-seeking and drug-taking behaviors. In addition, the agranular insular cortex (AI) is an important component of the neural circuits underlying drug-related memory reconsolidation. GABA_B_ receptors (GABA_B_Rs) are potential targets for the treatment of addiction, and baclofen (BLF) is the only prototypical GABA_B_ agonist available for application in clinical addiction treatment. Furthermore, ΔFosB is considered a biomarker for the evaluation of potential therapeutic interventions for addiction. Here, we used the morphine-induced conditioned place preference (CPP) paradigm to investigate whether postretrieval microinjections of BLF into the AI could affect reconsolidation of drug-reward memory, reinstatement of CPP, and the level of ΔFosB in mice. Our results showed that BLF infused into the AI immediately following morphine CPP memory retrieval, but not 6 h postretrieval or following nonretrieval, could eliminate the expression of a morphine CPP memory. This effect persisted in a morphine-priming–induced reinstatement test, suggesting that BLF in the AI was capable of preventing the reconsolidation of the morphine CPP memory. Our results also showed that the elimination of morphine CPP memory was associated with reduced morphine-associated ΔFosB expression in the longer term. Taken together, the results of our research provide evidence to support that GABA_B_Rs in the AI have an important role in drug-cue memory reconsolidation and further our understanding of the role of the AI in drug-related learning and memory.

## Introduction

Drug addiction is a serious public health concern worldwide and a chronic brain disorder molded by strong biosocial factors, leading to devastating consequences for individuals, families, and society (Volkow and Boyle, 2018; Padmanathan et al., 2019). However, there is currently no effective treatment for drug addiction (YY et al., 2019), especially relapses following abstinence, and drug cravings are often triggered by environmental cues that are repeatedly paired with the psychotropic drug (Torregrossa and Taylor, 2013). Therefore, attenuating the strength of cue-drug memories could reduce the number of factors that cause drug craving and relapse, which can help to treat drug addiction (Torregrossa and Taylor, 2013). Interestingly, impairing cue-drug memory reconsolidation, a short labile phase that can be induced once consolidated drug-associated memories are retrieved, is a generally accepted strategy aimed at reducing the intensity of cues to trigger drug-seeking and drug-taking behaviors (MT et al., 2019). Recent studies have shown that pharmacological interventions following the retrieval of drug-related cues could disrupt the reconsolidation of drug memories, inhibit conditioned place preference (CPP), and inhibit operant drug craving and relapse after withdrawal (Sanchez et al., 2010; Hasbi et al., 2017). However, most of the compounds used in these studies are used only in basic research and not in clinical studies, which may be a barrier to successful translation into clinical applications.

Drug craving depends critically on the function of the insular cortex (IC), which is critically involved in regulating interoception, and on the highest levels of the interoceptive system (Naqvi et al., 2007), which are involved in the perception of an individual's physical state, emotions, and needs, especially drug craving in those with drug addictions (Centanni et al., 2019). The IC is subdivided into the agranular insular cortex (AI) and granular insular cortex (GI) (Cocker et al., 2016; Gogolla, 2017), and they do not share the same functions and neural connections. In particular, the GI plays an important role in modulating visceral function and encodes primary aversive sensory information (Moraga-Amaro and Stehberg, 2012; Pushparaj and Le Foll, 2015). Because the AI is thought to maintain cognitive representations of interoceptive states associated with previous experiences, presumably including drug-related experiences, and combines these into a conscious affective state (Naqvi et al., 2014; Arguello et al., 2017), the AI is considered part of the high-order interoceptive cortex (Contreras et al., 2012). In addition, the AI is involved in cue-drug effect associations and is a key brain region for manipulating cue-drug–related memory reconsolidation (Contreras et al., 2012; Liu et al., 2016).

The existing literature suggests that GABA_B_ receptors (GABA_B_Rs) have been identified as a potentially therapeutic target for addiction (Chiamulera et al., 2017) because they have the effect of negatively modulating the reward, reinforcement, and reinstatement of drug seeking in the brain's reward circuit (Vlachou et al., 2011). Baclofen (BLF), a full GABA_B_R agonist, is the only specific GABA_B_ compound available for clinical research trials (Brebner et al., 2002). For a long time, ΔFosB has often been considered a molecular switch for addiction that helps initiate and then maintain an addicted state and a biomarker for evaluating the potential therapeutic interventions for addiction (Ruffle, 2014). However, whether intra-AI BLF has an effect on drug-related memory reconsolidation of CPP, a typical protocol for drug reward memory, has not been reported (Lin et al., 2014).

To address the above scientific questions in our study, we examined the effect of intra-AI infusions of the GABA_B_Rs agonist, BLF, on the reconsolidation of drug reward memory using the CPP paradigm, and the resulting memory alteration would be indicated by subsequent reduction in the time spent in the morphine-paired chamber. We hypothesized that in mice, reconsolidation of morphine CPP memory induced by a brief exposure to the conditioned stimulus is negatively modulated by GABA_B_Rs in the IC. From such hypothesis, we could derive a prediction that following re-exposure to the morphine-paired context, the activation of GABA_B_Rs in AI would interfere with the reconsolidation of morphine-cue memory.

## Materials and Methods

### Animals

Male C57BL/6J mice (12 weeks old, 23–28 g) were purchased from the Experimental Animal Center of Ningxia Medical University. These animals were housed under a 12-h light/dark cycle and constant room temperature (20–25°C) with access to water and rodent food ad libitum. The mice were housed in groups (4 to 5 per cage). The animal study was approved by the Animal Research Ethics Committee of Ningxia Medical University (2019-152). All efforts were made to minimize suffering and the number of animals used.

### Drugs and Antibodies

Morphine hydrochloride (specification: 10 mg/ml, First Pharmaceutical Factory of Shenyang, Shenyang, China) was diluted with sterile physiological saline and injected intraperitoneally (10 mg/kg) in a volume of 10 ml/kg before exposure to the morphine-paired context during the CPP training sessions. BLF (Sigma-Aldrich, St. Louis, MO, USA) was dissolved in sterile physiological saline. The BLF doses, microinjected into bilateral AI in mice, were chosen based on pilot experiments and recent research at the dose of 0.06 nmol/0.2 μl/side (Takahashi et al., 2010; Araki et al., 2016). Rabbit polyclonal antibodies against ΔFosB and rabbit monoclonal antibodies against c-Fos were purchased from Abcam (Cambridge, UK). Mouse monoclonal antibodies against β-actin and anti-mouse secondary antibodies were purchased from Zsbio Commerce Store (Beijing, China).

### Surgery and Intracranial Microinjection

Mice were anesthetized with sodium pentobarbital (40 mg/kg, i.p.) and had a normal body temperature maintained *via* a feedback-controlled heat blanket (TR-200, Safebio, Shanghai, China). Then, the anesthetized mice were transferred to a stereotaxic apparatus. Their heads were shaved and cleaned before making an incision. Following the incision and blunt separation of soft tissues to expose the skull, the placement of a stainless steel guide cannula (outer diameter (o.d.): 0.41 mm, inner diameter (i.d.): 0.25 mm; RWD Life Science Co., Ltd., Shenzhen, China) bilaterally 1 mm above the AI was determined according to the bregma. The stereotaxic coordinates of the AI were as follows: anterior/posterior (AP), +0.5 mm; medial/lateral (ML), ± 3.5 mm; and dorsal/ventral (DV), −4 mm. A hole was drilled in the skull, and the stainless steel guide cannula was bilaterally implanted in the AI. The guide cannulas were secured with three small screws and dental cement, and a capped stylet (o.d.: 0.20 mm; RWD Life Science Co., Ltd, Shenzhen, China) was inserted to prevent occlusion. The mice were handled every other day to reduce the stress of handling at the time of testing. After surgery, the animals were allowed to recover for one week. The capped stylet was removed, and a 32-gauge Hamilton microsyringe (volume: 0.5 μl, Mode 7000.5 KH SYR, Knurled Hub) attached to polyethylene tubing was inserted into the injection cannula (o.d.: 0.21 mm, i.d.: 0.11 mm; RWD Life Science Co., Ltd, Shenzhen, China). The other end of the tubing was connected to the Hamilton microsyringe placed into an infusion pump (CMA Microdialysis). BLF (0.06 nmol/0.2 μl) was microinjected into the AI in a 0.2-μl volume over 5 min (i.e., at the rate of 40 nl/min). The injection cannula was left in place for an additional 60 s after the injection before slowly removing it to allow the drug to completely diffuse. Then, the capped stylet was reinserted into the guide cannula.

### CPP Apparatus and Procedures

**Figure 1:**
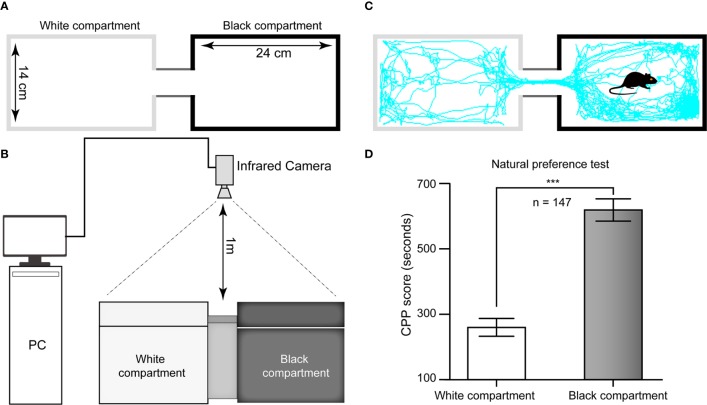
Schematic diagram of the CPP apparatus and histogram of natural preference. **(A)** Top view of the CPP chamber and its specifications. **(B)** Sketch of the ethological video tracking system. **(C)** Representative track of one mouse in our study during the 15-min period during the test in the Pre-C stage, suggesting that its natural preference is the black chamber. **(D)** All mice had a natural preference for the black compartment, i.e., the white chamber was nonpreferred, and the black chamber was preferred. AI, agranular insular cortex. ***p < 0.001, comparison of the staying time of mice between the black and white chambers.

The CPP apparatus comprised a rectangular plastic chamber separated by a guillotine door into two 24 cm × 14 cm × 30 cm compartments: one compartment had a smooth white floor and white walls, and the other component had a rough black floor and black walls. In brief, the floor texture and color of the two large chambers were different from each other to provide distinct tactile and visual cues paired with morphine or saline injections. This apparatus was situated in a dimly lit room. The tracking of the mice was monitored by an infrared video camera suspended approximately 1 m above the test arena. The time spent and distance traveled in each compartment were analyzed from the video data by a computerized video tracking system (SMART 3.0, Panlab, Spain, supported by RWD Life Science Co., Ltd, China) (**Figures 1A–C**).

The CPP was conducted using a biased protocol and based on prior literature with slight alterations (Wu et al., 2012). There were five different phases: Post-surgery (day −10 through day −3; D-10–D-3), Habituation (D-3–D-1), Pre-C (D0), Conditioning (D1–D8), and Post-C (D9). On the three habituation days, the animals were given 30 min in the apparatus to adequately become familiar with the experimental context and reduce stress, and they had access to both compartments of the CPP apparatus during the habituation phase. The next day, the individual baseline preference was determined. Each rodent was placed in the apparatus and allowed to freely explore the chambers for 15 min, the duration of the test session. The natural preference (Pre-C test) was tested after three days of habituation. As shown in **Figure 1D**, the white chamber was nonpreferred, and the black chamber was preferred in this study. During the morphine conditioning periods, the guillotine door was closed, and then the animal was assigned to receive morphine (10 mg/10 ml/kg, i.p.) in the nonpreferred compartment while physiological saline (10 ml/kg, i.p.) was administered in the preferred compartment. During the conditioning days, the mice were trained for 8 consecutive days (D1–D8) with alternating injections of morphine (10 mg/10 ml/kg, i.p.) in their nonpreferred compartment or saline (10 ml/kg, i.p.) in their preferred compartment. They were injected only once per day, with either morphine or saline. After each injection, the mice were placed in the morphine- or saline-conditioned chamber for 45 min before being returned to their home cages. One day after the last conditioning trial (D9), the postconditioning test (Post-C) was conducted in a drug-free state with the guillotine door removed.

The effective retrieval of the drug-related memory for consolidation is the precondition for the reconsolidation process. According to the preliminary experiments and previous studies, the morphine CPP memory was retrieved *via* a trial with the conditioned stimulus + unconditioned stimulus (i.e., CS + US) presented in which animals were given morphine (10 mg/kg, i.p.) and confined to the drug-paired compartment for 15 min (Wu et al., 2012). After this retrieval of the consolidated addiction-related memory, the effect of intra-AI BLF (0.06 nmol/0.2 μl/side) administration on memory reconsolidation was examined. CPP was tested at the following posttreatment (PT) times: 1 day (PT-D1) or 14 days (PT-D14) after the administration of BLF. At posttreatment day 15 (PT-15D), the mice received a priming injection of morphine (5 mg/kg, i.p.), and the CPP test was immediately performed after the priming injection (Priming) (Wright et al., 2019).

### Experimental Design

#### Experiment 1: The Effect of Morphine Reward Memory Retrieval on Neural Activity in the IC

**Figure 2:**
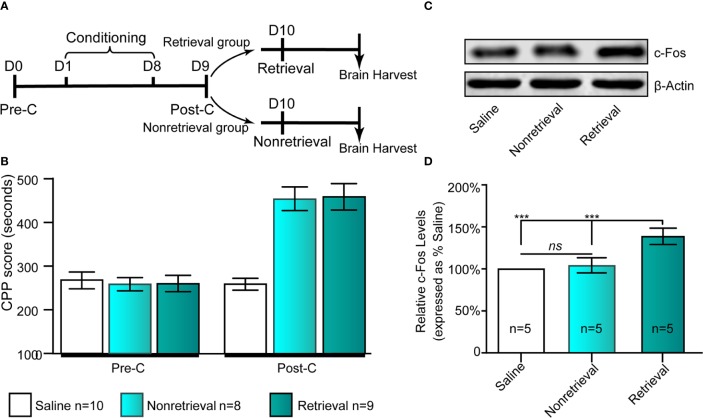
Influence of the retrieval of drug-cue memory on the expression of c-Fos in the AI. **(A)** The experimental timeline of the behavioral procedure. **(B)** CPP scores of the saline, nonretrieval, and retrieval groups in the Pre-C and Post-C stages. **(C)** Western blot analysis of the relative protein content of c-Fos in the saline, nonretrieval, and retrieval groups. **(D)** Semiquantitative analysis of the relative levels of c-Fos by densitometric analysis (n = 5 mice per group, one-way ANOVA). ***p < 0.001; ns, no significance. Data are represented as the mean ± SEM.

Given that c-Fos has been used as a neural activity marker for a long time (Chung, 2015), we could assess neuronal activation in the IC following drug-cue memory retrieval by measuring the protein level of c-Fos in the IC. As shown in **Figures 2A, B**, the retrieval and nonretrieval groups were trained in the CPP for 8 days except for the saline group, which was intraperitoneally injected with physiological saline only. On day 9, all mice were tested for CPP (Post-C). On day 10, the retrieval group mice, rather than the nonretrieval group (i.e., without retrieval), were retrieved *via* presentation of CS + US, and then the mouse brains were harvested for c-Fos Western blotting (**Figure 2C**).

#### Experiment 2: The Effect of Bilateral Intra-AI BLF Immediately Following Memory Retrieval on Memory Reconsolidation

**Figure 3:**
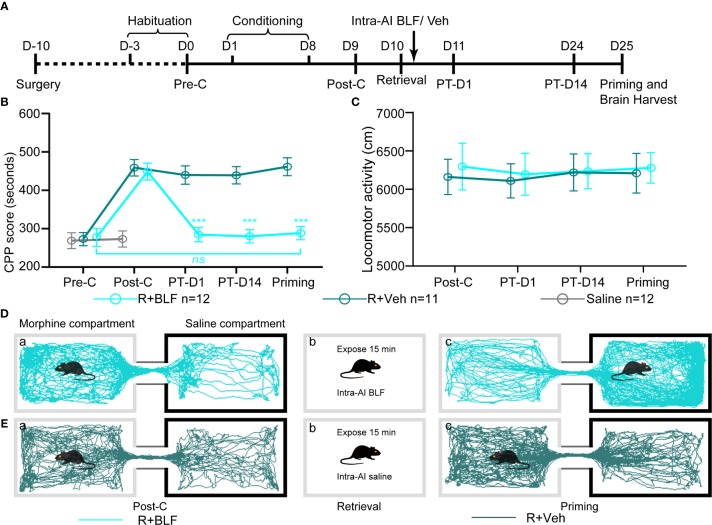
Effects of intra-AI vehicle and 0.06 nmol/side BLF on the reconsolidation of CPP following reward memory retrieval. **(A)** Timeline of experimental procedure. **(B)** Intra-AI 0.06 nmol/side BLF following reward memory retrieval could eliminate the established consolidated CPP for more than 14 days by disrupting its reconsolidation. *** represents the R + BLF group vs. R + Veh group, *p* < 0.001, at the same time point. *ns* represents no significant difference. **(C)** There was no significant difference in the locomotor activity between the two groups. **(D**, **E)** Representative tracks of one mouse in the R + BLF group and the R + Veh group during the 15-min period of the test at the Post-C stage (**D**-a, **E**-a) and Priming stage (**D**-c, **E**-c), respectively. Furthermore, panels (**D**, **E**)-a), show that the mouse successfully established the CPP, that is, preferred the morphine compartment. Panels (**D**, **E**-b), show that it was exposed to the morphine compartment for 15 min and then administered 0.06 nmol/side BLF or 0.2 μl/side saline into the AI. Panel (**D**-c), shows that the mice in the R + BLF group no longer preferred the morphine chamber. In contrast, the mice in the R + Veh group still preferred the morphine chamber. Cyan tracks: R + BLF mouse; DarkSlateGray tracks: R + Veh mouse; R + BLF = Intra‐AI 0.06 nmol/0.2 μl/side BLF following reward memory retrieval; R + Veh = Intra‐AI 0.2 μl/side 0.9% sterile saline following the memory retrieval; PT-D1 = 1 day postdrug treatment; PT-D14 = 14 days postdrug treatment.

The retrieval + intra-AI 0.2 μl/side vehicle (R + Veh) and retrieval + intra-AI 0.06 nmol/side BLF (R + BLF) groups were trained in the CPP for 8 days except for the saline group, which was intraperitoneally injected with physiological saline only. On day 9, all mice were tested for CPP (Post-C). On day 10, the R + Veh and R + BLF groups were injected with morphine and then re-exposed to the morphine-paired chamber for 15 min, receiving bilateral BLF (0.06 nmol/side) or vehicle (0.2 μl) microinjections into the AI immediately after memory retrieval. On day 11 and day 24, all mice were retested for CPP (PT-D1 and PT-D14, respectively). On day 25, the mice in the R + Veh and R + BLF groups received a priming injection of morphine (5 mg/kg, i.p.), and the CPP test was immediately performed after the priming injection (Priming). Meanwhile, during the behavioral testing session above, the distance that each mouse moved (i.e., locomotor activity) during the 15 min was recorded. After all the behavioral tests were completed, the mice were anesthetized, and their brains were harvested for ΔFosB Western blotting and immunofluorescence experiments (**Figure 3A**).

#### Experiment 3: The Effect of Bilateral Intra-AI BLF Without Memory Retrieval on the Expression of a Morphine CPP Memory

The nonretrieval+0.2 μl/side vehicle (NR + Veh), nonretrieval+intra-AI 0.06 nmol/side BLF (NR + BLF), and saline groups followed the same procedures as described for experiment 1 with the exception of day 10, during which the mice in the NR + Veh and NR + BLF groups were not re-exposed to the morphine-paired chamber and received bilateral BLF (0.06 nmol/0.2 μl/side) or vehicle (0.2 μl/side) microinjections into the AI without the reward memory retrieval (i.e., the administration of BLF in their home cage). After all behavioral tests were completed, the mice were anesthetized, and their brains were harvested for the ΔFosB Western blotting analysis (**Figure 6A**).

#### Experiment 4: The Effect of Bilateral Intra-AI BLF 6 h After Memory Retrieval on the Expression of a Morphine CPP Memory

The same procedure as experiment 1 was performed 6 h after retrieval+0.2 μl/side vehicle (6hR + Veh) 6 h after retrieval + intra-AI 0.06 nmol/side BLF (6hR + BLF) and with the saline group with the exception of day 10, during which the mice in the 6hR + Veh and 6hR + BLF groups were injected with morphine and re-exposed to the morphine-paired chamber for 15 min and then received bilateral BLF (0.06 nmol/0.2 μl/side) or vehicle (0.2 μl/side) microinjections into the AI 6 h after reward memory retrieval. After all behavioral tests were completed, the mice were anesthetized, and their brains was harvested for ΔFosB Western blotting analysis (**Figure 8A**).

#### Experiment 5: The Effect of Bilateral Intra-AI BLF on Drug-naive Mice

Following the Pre-C test on day 1, during the saline conditioning periods the mice were assigned to receive saline (10 ml/kg, i.p.) in both the nonpreferred and preferred compartments and were confined to the corresponding compartment for 45 min for eight consecutive days. Post-C was performed in a drug-free test (15 min). On day 10, the mice were re-exposed to the nonpreferred chamber (i.e., white chamber) for 15 min and then received bilateral microinjections of BLF (0.06 nmol/side) into the AI. We used a drug-free test to assess the effect of bilateral intra-AI BLF on drug-naive mice after 1 day (PT-D1) (**Figure 9A**).

### Immunofluorescent Labeling of ΔFosB in Mouse Brain Sections

The mice were fixed with 4% paraformaldehyde after anesthesia. Their brains were carefully removed and fixed in 4% paraformaldehyde for 6 h. Then, the specimens were placed in 30% sucrose (a cryoprotectant), embedded into optimal cutting temperature compound (OTC), frozen with dry ice and cut into 10-µm coronal sections. Following this, the tissue sections were treated with 3% hydrogen peroxide and incubated in 3% bovine serum albumin (BSA, Sigma-Aldrich, St. Louis, MO, USA) for 60 min followed by incubation at 4 °C overnight with the primary rabbit anti-FosB antibody (1:500). The next day, the sections were subsequently incubated with goat anti-rabbit IgG H&L (Alexa Fluor^®^ 488, Abcam, ab150077) (1:500) for 1 h at room temperature after washing in PBS three times and then washed and counterstained with DAPI (ZLI-9557, Zsbio Commerce Store, Beijing, China). Ultimately, images were captured with a Leica DM6 fluorescence microscope (Leica, Germany), and the fluorescence intensity was statistically compared between treatments or groups using ImageJ according to the literature, which lists specific operation methods and approaches (Jensen, 2013).

### Western Blot Analysis

Western blot was performed according to previous literature from our lab with slight alterations (Gao et al., 2019). The IC, which lies exposed on the lateral surface of the hemisphere, mostly 1 mm above the rhinal fissure in mice (Gogolla, 2017), was divided from the freshly harvested mouse brain and subsequently homogenized on ice using a Bullet Blender (Next Advance, Inc., Troy, NY, USA) in lysis buffer from the Total Protein Extraction Kit (KGP2100, KeyGEN Biotechnology Co., Ltd, Jiangsu, China) containing phosphatase inhibitors, protease inhibitors, and phenylmethanesulphonyl fluoride (PMSF). The IC was centrifuged for 15 min at 14,000 g at 4 °C, and the protein concentrations were determined *via* a bicinchoninic acid kit (BCA kit; KeyGen, Nanjing, China). Before boiling at 100 °C for 5 min to denature the proteins using a metal bath, total protein extraction was combined with a sodium dodecyl sulfate polyacrylamide gel electrophoresis (SDS-PAGE) loading buffer. Next, equal amounts of protein according to their concentrations from each group were separated by 10% SDS-PAGE, transferred onto a 0.45-μm polyvinylidene fluoride (PVDF) membrane (Millipore, U.S.A.) by wet transfer (300 mA, 1 h) and blocked for 1 h with 5% skim milk powder (Becton, Dickinson and Company, U.S.A.) at room temperature. Then, the membrane was incubated with primary antibodies, anti-FosB antibody (1:10000, Abcam, ab184938), and anti-β-actin (1:2000, Beijing Zhongshan Golden Bridge Biotechnology CO., LTD., TA-09), at 4°C overnight. After washing 5 times (5 min each) with TBST, the corresponding secondary antibodies [goat anti-mouse (1:10000, Abcam, ab216776) or goat anti-rabbit (1:10000, Abcam, ab216777)] were added for 1 h at room temperature. Subsequently, the Western blotting results were analyzed using the Odyssey scanner (Li-Cor Bioscience, USA). We used normalization method for the western blot quantification with the aim of minimizing effects arising from variations in experimental errors. In detail, the stained blot was imaged, a rectangle was drawn around the target protein(ΔFosB/FosB/c-fos) or internal controls protein (β-actin) in each lane, and the gray values inside the rectangle were measured with Image J software. The relative value of each of the target protein levels was equal to the gray values of each target protein divided by the corresponding internal controls. In this way, we obtained the relative gray values of the saline group, the experimental group, and the corresponding vehicle control group. Then the relative gray values of the three groups divided by the saline group. Finally, the normalizing Western blot data of the target protein content in each group was obtained.

### Cannula Verification

**Figure 4:**
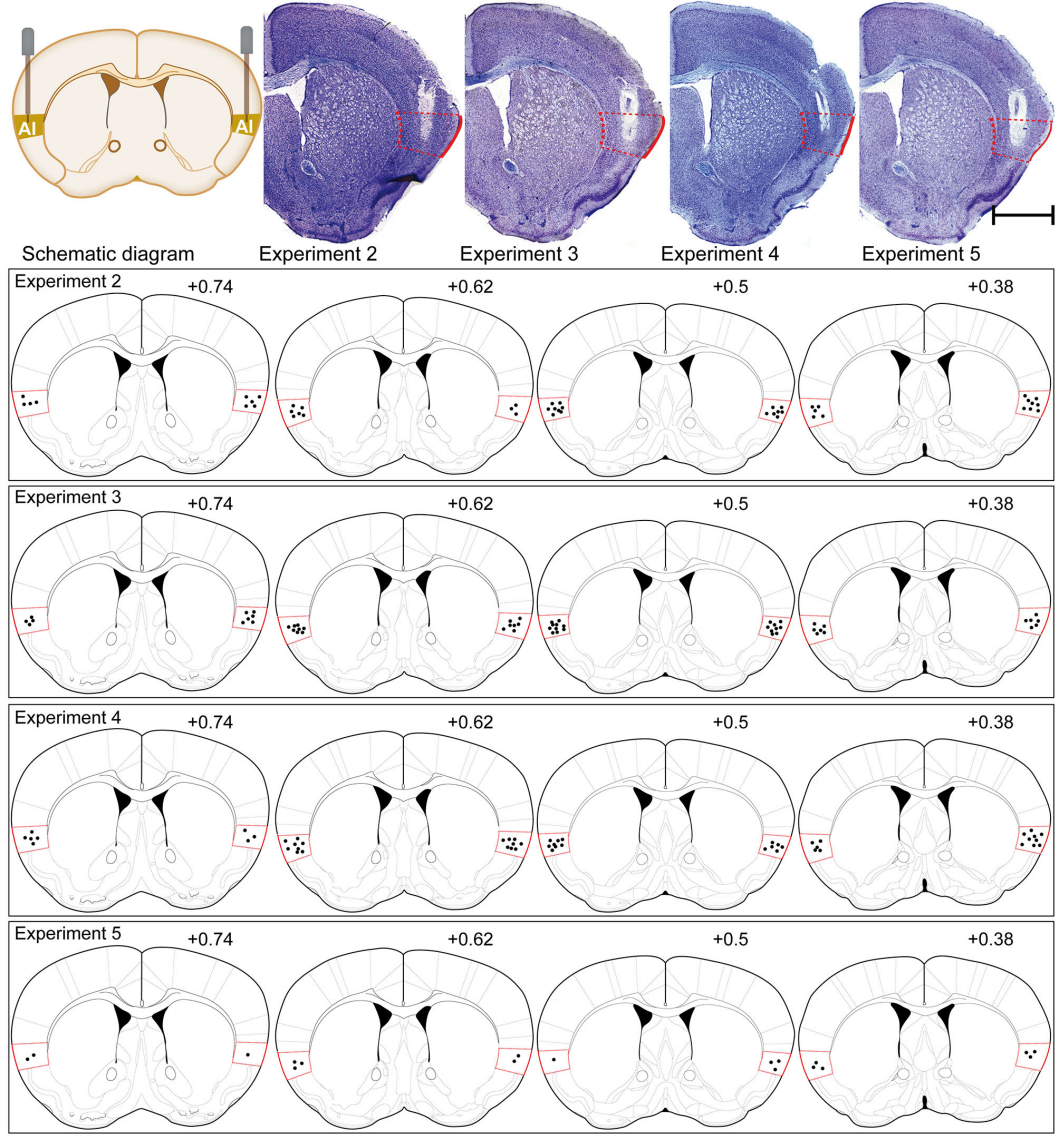
Schematic illustrations and representative photomicrographs of the intracranial cannula infusion sites in the AI with cresyl violet staining for each behavioral pharmacology experiment. Numbers besides the sections indicate anterior distance from bregma in millimeters. Data are reconstructed from the Paxinos and Franklin atlas (Konsman, 2003). Scale bar: 1 mm.

Approximately each subgroup needing to be placed in injection cannula had a total of 16 to 20 mice before verifying the injection cannula. They were all established with the CPP paradigm and operated in accordance with the procedures of each subgroup. After the behavioral studies were completed, these animals were subjected to cannula verification. Cannula verification is divided into two types, one for Nissl staining (8–10 mice) and one for their brains removed fresh (8–10 mice). Only the placement of injection cannula was accurately placed into AI for statistical analysis of behavior and for the next molecular biology experiment. Brains from implanted animals were either fixed and then removed or removed fresh, that is, animals assigned to immunofluorescence were perfused and verified with Nissl staining, and those assigned to western blot had their brains removed fresh. Specifically speaking, at the end of all behavioral tests, the mice assigned to immunofluorescence were sufficiently anesthetized with sodium pentobarbital and transcardially perfused with heparinized 0.9% saline until the fluid flowing out through the heart showed clear saline without bleeding, and then the brains were fixed with 4% paraformaldehyde. The mouse brains were gently removed and post-fixed in the same fixative for 6 h. Following, mice brains were coronally sliced with a microtome, and the slices thickness were 25-μm for Nissl staining when cutting to the AI injection cannula trace. When the brain slices just did not show the AI injection cannula trace, adjust the slice thickness to 10-μm for immunofluorescence experiments. The locations of the typical cannula tips were shown in **Figure 4**. In addition, the method of determining the end position of the injection cannula in the animal brain tissue for the Western blot experiment was as follows: following the removal of the mice brains, they were placed in a Brain Matrix Mouse (68713, RWD Life Science Co., Ltd., Shenzhen, China). A brain slice containing an AI injection cannula trace with a thickness of approximately 0.5 mm was cut, following placed on a glass slide. The position of the end of the injection cannula was determined using an optical microscope with reference to the Paxinos and Franklin atlas (Konsman, 2003). The IC was divided from the remaining brain tissue with a glass dissecting needle for Western blot analysis. Only the mice with cannulas correctly placed included in the data analysis.

### Statistical Analysis

The CPP score was defined as the time (in seconds) spent in the morphine-paired chambers during CPP testing and analyzed by two-way repeated measure analyses of variance (two-way rmANOVA) with between-subjects factors of drug treatment and within-subjects factors of test session (Pre-C versus Post-C; Post-C versus PT-D1, PT-D14, and Priming). *Post hoc* analyses of significant effects were performed using the Bonferroni test. Student's t test was used to determine statistical significance in order to make a comparison between two groups. For three or more groups of samples, the statistical significance was analyzed with one-way ANOVA followed by Tukey's *post hoc* analysis. Data were processed by commercially available software: SPSS 22.0 (SPSS Inc., Chicago, IL, USA) and GraphPad Prism v8.0 (GraphPad Software, Inc.). The values are presented as the mean ± SEM.

## Results

### Cannula Verification

The placements of injection cannula needle tips targeted to the AI were verified by postmortem histological verification according to the Paxinos and Franklin atlas (Konsman, 2003) (**Figure 4**). Overall, 43 of 129 mice were discarded because their cannula placements were outside the AI. As the cannula placements of 86 mice were accurately placed in the AI, their behavioral and molecular biology data were included in our study.

### Natural Preference Test

For the 147 mice in this study, the time spent in the black chamber was significantly longer than that in the white chamber in the Pre-C stage (*t*_(292)_= 43.93, *p* < 0.001, Student's t test, **Figure 1D**), indicating that all mice had a natural preference for the black chamber, i.e., the white chamber was nonpreferred, and the black chamber was preferred.

### Experiment 1: Drug-Cue Memory Retrieval Activates the IC

To study the influence of morphine reward memory reconsolidation on the activity of the IC, we examined the expression of c-Fos, a molecular marker of neuronal activation (Curran and Morgan, 1995; Chen et al., 2019), following retrieval of morphine-cue memory (i.e., CS + US) in the IC using Western blotting (**Figures 2A, B**). We could see that the expression of c-Fos in the IC significantly increased in the retrieval group (F_(2,__14)_ = 29.28, *p* < 0 .001, one-way ANOVA followed by Tukey's multiple comparison test, **Figures 2C, D**). Because the mice also experienced morphine conditioning in the retrieval group, we examined the change in the expression of c-Fos in the IC in nonretrieval group (only morphine conditioning without retrieval) to exclude the activating effects of drug conditioning. The results showed that compared with the saline group, nonretrieval mice had no significant influence on the expression of c-Fos in the IC (*t*_(8)_= 1.91, *p* =0.09, Student's t test, **Figure 2D**). In brief, the retrieval of morphine-cue memory can activate the neurons of the IC in mice.

### Experiment 2: Intra-AI BLF Following Memory Retrieval Disrupted the Reconsolidation of the Morphine CPP and Prevented the Reinstatement of the CPP

The two-way ANOVA revealed that all morphine conditioning groups acquired a CPP (n = 94, Pre-C: 268 ± 70 s; Post-C: 453 ± 81 s) compared with the saline group (n = 53, Pre-C: 268 ± 72 s; Post-C: 270 ± 66 s) after conditioning [F_(1,__145)_ = 58.25, *p* < 0.001]. The CPP scores did not differ among any morphine conditioning groups before the intra-AI BLF or saline microinjections [F _(7,__86)_ = 0.23 *p* = 0.85] (**Figures 3B, D, E**). The effect of intra-AI BLF following memory retrieval on the reconsolidation of the CPP is shown in **Figures 3B, D, E**. With treatment (postretrieval intra-AI BLF or saline) as the between-subjects factor and test session (Post-C, PT-D1, PT-D14, Priming) as the within-subjects factor, the two-way rmANOVA revealed a significant effect of treatment (F_(1,__22)_ = 18.81, *p* < 0.001), test point (F_(3,22)_ = 144.35, *p* < 0.001), and treatment × test point interaction (F_(3,22)_ = 105.69, *p* < 0.001). In addition, the Bonferroni *post hoc* analyses confirmed that compared with the R + Veh group, the CPP scores in the R + BLF group were decreased significantly at PT-D1, PT-D14, and the Priming test point. Furthermore, the CPP scores for the R + BLF group during the priming test did not differ from those during Pre-C (Pre-C 277 ± 81 s versus Priming 288 ± 60 s, *t*_(22)_= 0.38, *p* =0.71, Student's t test), indicating that morphine priming did not reinstate the CPP. In summary, these results suggest that intra-AI administration of 0.06 nmol/side BLF following reward memory retrieval could eliminate the established consolidated CPP for more than 14 days and inhibit the reinstatement of CPP by disrupting its reconsolidation. Moreover, as depicted in **Figure 3C**, the locomotor activity of mice was not significantly different between the R + BLF and R + Veh groups (treatment: F _(1,__22)_ = 0.06, *p* = 0.81; test point: F_(3,22)_ = 0.28, *p* = 0.69; interaction F_(3,22)_ = 0.17, *p* = 0.65), which indicated that administration of 0.06 nmol/side BLF into the AI following memory retrieval had no effect on the locomotor activity of mice.

**Figure 5:**
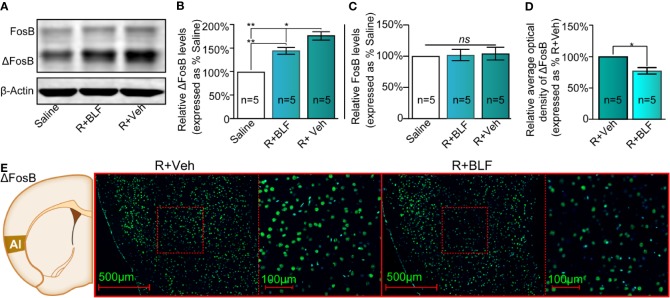
Effects of intra-AI BLF on the protein levels of ΔFosB following reward memory retrieval using Western blot and immunofluorescence analyses. **(A)** Western blot analysis of the relative protein content of ΔFosB and FosB in the saline, R + BLF and R + Veh groups. **(B, C)** Semiquantitative analysis of the relative levels of ΔFosB and FosB by densitometric analysis (n = 5 mice per group, one-way ANOVA). **(D)** Semiquantitative analysis of the relative levels of ΔFosB by densitometric analysis in the R + BLF and R + Veh groups (n = 5 per group). **(E)** Representative immunofluorescence images of ΔFosB in the AI from the R + BLF and R + Veh groups in the left panel. The corresponding scope of the magnified images right is marked by red squares. Data are presented as the mean ± SEM analyzed by one‐way ANOVA. * and ** represent *p* < 0.05 and *p* < 0.01, respectively. ns represents no significant difference.

At the end of the priming test, the mouse brains were harvested, and Western blotting or immunofluorescence assays were performed. The saline, R + Veh, and R + BLF groups showed significantly different relative protein content of ΔFosB by Western blotting (F_(2,__14)_ = 52.82, *p* < 0.0001, one-way ANOVA, **Figures 5A, B**), and then Tukey's multiple comparison *post hoc* analysis showed that the protein content of ΔFosB was significantly different between the R + Veh and R + BLF groups (*p* = 0.01), which indicated that 15 days following postretrieval activation of GABA_B_Rs in the AI *via* intra-AI BLF could decrease the protein level of ΔFosB. However, there was no significant difference in the relative protein content of FosB among the three groups (F_(2,__14)_ = 0.41, *p* = 0.67, one-way ANOVA, **Figure 5C**). As shown in **Figures 5D, E**, the fluorescence intensity significantly decreased in the R + BLF group compared with the R + Veh group (*t*_(8)_= 2.78, *p* = 0.03, Student's t test).

### Experiment 3: Intra-AI BLF Without Memory Retrieval Had No Effect on the Expression of a Morphine CPP Memory

**Figure 6:**
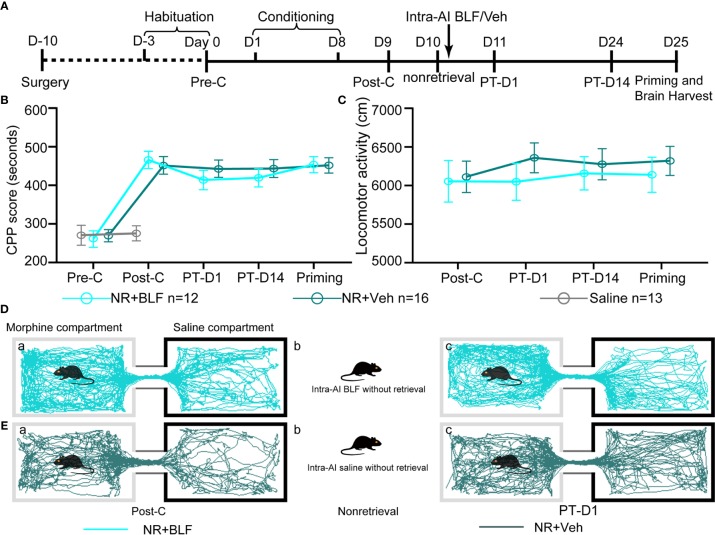
Effects of intra-AI 0.06 nmol/side BLF and 0.2 μl/side vehicle on the conditioned response without reward memory retrieval. **(A)** Timeline of the experimental procedure. **(B, C)** Intra-AI BLF without reward memory retrieval has no effect on the conditioned response and locomotor activities. **(D, E)** Representative tracks of one mouse in the NR + BLF group and the NR + Veh group in the Post-C stage and PT-D1 stage. Furthermore, panels (**D**-a and **E**-a), show that the mouse successfully established the CPP, that is, preferred the morphine compartment. Panels (**D**-b and **E**-b), show that it was not exposed to the morphine compartment and administered directly 0.06 nmol/side BLF or 0.2 μl/side saline into the AI. Panels (**D**-c and **E**-c) show that its preference did not change; that is, it still preferred the morphine chamber. Cyan tracks: R + BLF mouse; DarkSlateGray tracks: R + Veh mouse; NR + BLF = Intra‐AI 0.06 nmol/0.2 μl/side BLF without reward memory retrieval; NR + Veh = Intra-AI 0.2 μl/side 0.9% sterile saline without memory retrieval; PT-D1 = 1 day postdrug treatment; PT-D14 = 14 days postdrug treatment.

To determine whether intra-AI BLF indiscriminately impairs CPP reconsolidation, the administration of 0.06 nmol/side BLF into the AI was performed in their home cage without the morphine reward memory retrieval, which is an essential step to investigate whether the BLF-induced disappearance of the CPP was mediated by the disruption of its reconsolidation. As shown in **Figures 6B, D, E**, with treatment (nonretrieval intra-AI BLF and saline) as the between-subjects factor and test session (Post-C, PT-D1, PT-D14, Priming) as the within-subjects factor, although the two-way rmANOVA of mCPP scores revealed a significant main effect of test point (F_(3,26)_ = 23.84, *p* < 0.001), no significant difference in treatments (F_(1,26)_ = 0.09, *p* = 0.41), and interaction effect (F_(3,__26)_ = 4.7, *p* = 0.07) was detected. The results showed that administration of BLF into the AI without memory retrieval failed to affect the conditioned response, which suggested that the persistent amnestic effect of morphine CPP memory following just a single intra-AI microinfusion of BLF was retrieval-dependent, adding support to the reconsolidation hypothesis. Furthermore, the locomotor activity of mice was not significantly different between the NR + BLF and NR + Veh groups (treatment: F _(1,__26)_ = 0.61, p = 0.65; test point: F_(3,26)_ = 1.16, *p* = 0.07; interaction F_(3,26)_ = 0.35, *p* = 0.12, two-way rmANOVA, **Figure 6C**), which indicated that administration of 0.06 nmol/side BLF into the AI without memory retrieval had no effect on locomotor activity.

**Figure 7:**
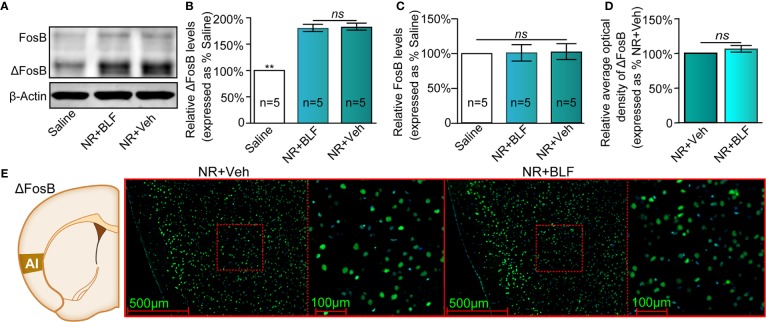
Effects of nonretrieval intra-AI BLF on the protein levels of ΔFosB using Western blot and immunofluorescence analyses. **(A)** Western blot analysis of the relative protein content of ΔFosB and FosB in different groups. **(B, C)** Semiquantitative analysis of the relative levels of ΔFosB and FosB by densitometric analysis (n = 5 mice per group, one-way ANOVA). **(D)** Semiquantitative analysis of the relative levels of ΔFosB by densitometric analysis in the NR + BLF and NR + Veh groups (n = 5 per group). **(E)** Representative immunofluorescence images of ΔFosB in AI from the NR + BLF and NR + Veh groups in the left panel. The corresponding scope of the magnified images right is marked by red squares. Data are presented as the mean ± SEM analyzed by one‐way ANOVA. ** represents p < 0.01, compared with the saline group. ns represents no significant difference.

The saline, NR + Veh and NR + BLF groups showed significantly different relative protein content of ΔFosB by Western blotting (F_(2,__14)_ = 85.75, *p* < 0.0001, one-way ANOVA, **Figures 7A, B**). Tukey's multiple comparison *post hoc* analysis showed that the protein content of ΔFosB was no significant difference between the NR + Veh and NR + BLF groups (*p* = 0.87), which indicated no effect on the level of ΔFosB at 15 days after administration of 0.06 nmol/side BLF into the AI without memory retrieval. In addition, there was no significant difference in the relative protein content of FosB among the three groups (F_(2,__14)_ = 0.35, *p* = 0.72, one-way ANOVA, **Figure 7C**). As shown in **Figures 7D, E**, there was no significant difference in the fluorescence intensity between the NR + BLF group and the NR + Veh group (*t*_(8)_= 1.9, *p* = 0.09, Student's t test).

### Experiment 4: Intra-AI BLF 6 h After Memory Retrieval Had No Effect on the Expression of a Morphine CPP Memory

**Figure 8:**
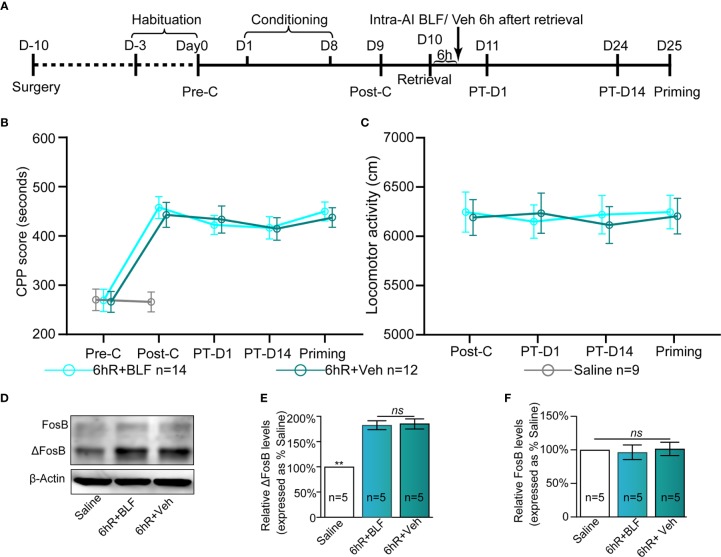
Effects of intra-AI 0.06 nmol/side BLF and 0.2 μl/side vehicle on the conditioned response 6 h after reward memory retrieval. **(A)** Timeline of the experimental procedure. **(B, C)** Intra-AI BLF 6 h after reward memory retrieval has no effect on the conditioned response and locomotor activities. **(D)** Western blot analysis of the relative protein content of ΔFosB and FosB in different groups. **(E, F)** Semiquantitative analysis of the relative levels of ΔFosB and FosB by densitometric analysis (n = 5 mice per group, one-way ANOVA). Data are presented as the mean ± SEM. 6hR + BLF = Intra‐AI 0.06 nmol/ side BLF 6 h after retrieval; 6hR + Veh = Intra‐AI 0.2 μl/side 0.9% sterile saline 6 h after retrieval. ** represents p < 0.01. ns represents no significant difference.

There is evidence to show that the processes underlying morphine reward memory reconsolidation occur over a finite duration that is less than 6 h after reactivation (Li et al., 2015). To definitively conclude that reconsolidation is impacted by BLF infusions, we considered including an experiment that received infusions 6 h after memory reactivation. With treatment (post-retrieval 6 h intra-AI BLF and saline) as the between-subjects factor and test session as the within-subjects factor (Post-C, PT-D1, PT-D14, and Priming), although the two-way rmANOVA of mCPP scores revealed a significant main effect of test point (F_(3,24)_ = 20.57 *p* < 0.001), no significant difference in treatments (F_(1,24)_ = 0.03, *p* = 0.49), and interaction effect (F_(3,24)_ = 5.25, *p* = 0.09; **Figure 8B**) was detected. The results showed that administration of 0.06 nmol/side BLF into the AI 6 h after retrieval failed to affect the conditioned response, ultimately indicating that reconsolidation is impacted definitively by BLF infusions. Furthermore, the locomotor activity of the mice showed an insignificant difference between the 6hR + Veh and 6hR + BLF group (treatment: F_(1,__24)_ = 0.21, *p* = 0.74; test point: F_(3,24)_ = 0.474, *p* = 0.41; interaction F_(3,24)_ = 1.07, *p* = 0.09; two-way rmANOVA, **Figure 8C**), indicating that administration of 0.06 nmol/side BLF into the AI 6 h after retrieval had no effect on the locomotor activity of mice.

The saline, 6hR + Veh, and 6hR + BLF groups showed significantly different relative protein content of ΔFosB by Western blotting (F_(2,__14)_ = 109.64, *p* < 0.0001, one-way ANOVA, **Figures 8D, E**). Tukey's multiple comparison *post hoc* analysis showed that the protein content of ΔFosB was no significant difference between the 6hR + Veh and 6hR + BLF groups (*p* = 0.91), which indicated no effect on the level of ΔFosB at 15 days after administration of 0.06 nmol/side BLF into the AI 6 h after retrieval. In addition, there was no significant difference in the relative protein content of FosB among the three groups (F_(2,__14)_ = 0.16, *p* = 0.86, one-way ANOVA, **Figure 8F**).

### Experiment 5: Intra-AI BLF Had No Effect on Drug-Naive Mice

**Figure 9:**
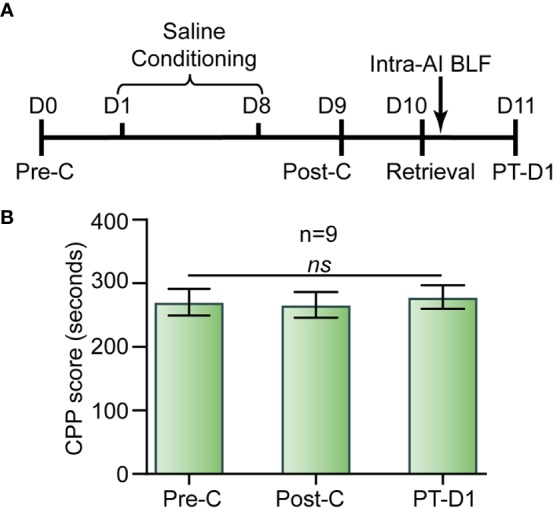
Effects of the administration of BLF into the AI in drug-free mice. **(A)** Timeline of experimental procedure. **(B)** BLF microinjection into the AI had no effect on CPP. Data are presented as the mean ± SEM. ns represents no significant difference.

As shown in **Figure 9**, there was no significant difference between Pre-C (270 ± 65 s) and Post-C (266 ± 60 s) in the drug-naive mice group (*t*_(8)_= 0.42, *p* = 0.69, paired samples *t*-test), indicating that saline conditioning had no effect on natural preference. Then, 0.06 nmol/side BLF was microinjected into the AI immediately after re-exposure to the nonpreferred chamber (i.e., white chamber) for 15 min, and the CPP scores showed no significant difference between Post-C and PT-D1 (278 ± 63 s) (*t*_(8)_ = 0.74, *p* = 0.48, paired samples *t*-test). Our results indicated that a microinjection of BLF into the AI immediately following re-exposure to the nonpreferred chamber had no aversive or rewarding effect on the drug-naive mice.

## Discussion

Reconsolidation is usually characterized as a process that allows memory strength to be adjusted or updated by integrating new information into the original memory during a limited period after reactivation or retrieval, and it has aroused great interest in disruption of the maladaptive memories of drug addiction (Exton-McGuinness et al., 2019; Rich et al., 2020). Our study observed the effects of intra-AI BLF on the reconsolidation of CPP in mice *via* behavioral pharmacology methods. The main results of our study are as follows: Postretrieval BLF infusions into the AI resulted in the elimination of the expression of a morphine CPP memory. This effect persisted in a morphine-priming-induced reinstatement test, suggesting that the BLF in the AI was capable of preventing the reconsolidation of the morphine CPP memory. In addition, this effect was observed only if administration occurred immediately postretrieval and not when BLF was administered in the absence of retrieval or 6 h postretrieval. The molecular mechanism of the disappearance of the CPP induced by BLF may be related to the decrease in ΔFosB levels in the AI.

c-Fos is an immediate early gene (IEG) marker commonly used to identify neurons that are activated after a stimulus or behavioral conditioning (Chung, 2015; Xu et al., 2019). Recently, it has been reported that the neuronal c-Fos protein expression levels in the nucleus accumbens (NAc) are elevated following cocaine context-associated reward memory retrieval, which demonstrates that NAc is activated during cocaine context-associated reward memory retrieval (Wang et al., 2014). However, given that the insula and NAc have been shown to be critical neural substrates for addiction (Naqvi et al., 2014; Droutman et al., 2015), whether IC neurons are activated following reward memory retrieval has not been reported. This study provides evidence that the c-Fos protein expression levels are significantly increased following morphine reward memory retrieval, which suggests that neurons in the IC are activated and are the crucial bases of morphine cue-associated reward memory.

More than one hypothesis could explain the BLF-induced disappearance of the CPP expression in our study. First, the loss of CPP could be simply through the administration of intra-AI BLF per se rather than an impairment in the memory reconsolidation of CPP combined with the intra-AI BLF. However, this explanation looks to be untrue for the reason that there was no effect on the expression of CPP in the NR + BLF and 6hR + BLF mice, which all experienced the same morphine conditioning and test procedure but received an injection of 0.06 nmol/side BLF into the AI without memory retrieval and 6 h after memory retrieval, respectively. Additionally, our results showed that the injection of BLF into the AI had no effect on locomotor activities, which suggested that the BLF-induced disappearance of CPP was not due to its sedative side effects. These results indicated that BLF disrupted the reconsolidation of CPP and that the loss was not simply an effect of BLF administration itself.

The second possible hypothesis is that postretrieval intra-AI BLF enabled the CPP memory to be extinguished. Before determining whether this hypothesis is correct, it is necessary to understand the difference between the extinction of morphine CPP memory and the elimination of morphine CPP memory caused by pharmacological interference with morphine reward memory reconsolidation. Chronic use of psychoactive drugs causes the formation of pathological memories of environmental cues associated with the drug use experience (Torregrossa and Taylor, 2013). Indeed, given that associations between drug (unconditioned stimulus, US) and drug-related cues (conditioned stimulus, CS) play an important role in drug addiction and relapse, exposure to just the cues is sufficient to induce drug seeking and relapse (YY et al., 2019). Therefore, reducing the intensity of cue-drug memories has gradually become a focus of attention in the treatment of addiction and the prevention of drug craving and relapse. The extinction of drug CS by nonreinforced exposure and/or abolishing cue-drug memory reconsolidation are two strategies to reducing the strength of drug seeking and prevent relapse (Torregrossa and Taylor, 2013). However, the limitations of extinction as a technique to prevent the relapse of maladaptive behavior are obvious; in other words, the recovery of extinction is frequent following the passage of time (spontaneous recovery) and following presentation of the US (reinstatement). The cause of the repeated recovery of extinction may be that it does not erase or modify the original CS-US associative memory to the greatest extent (Dunsmoor et al., 2015). In contrast, previously maladaptive reward memories underpinning addiction can be disrupted or erased pharmacologically following retrieval of the memory trace (Exton-McGuinness et al., 2019). Therefore, reconsolidation-based therapeutic strategies for addictive drug-seeking and preventing relapse maybe more promising than enhancing the consolidation of extinction learning. Hence, according to the above discussion, the second hypothesis is also wrong because CPP extinction involves temporal inhibition of the expression of the established CPP, which could be primed by the unconditioned stimulus (i.e., morphine injection, 5 mg/kg i.p.) to reinstate the original CPP (Khaleghzadeh-Ahangar and Haghparast, 2019). Nevertheless, in this study reinstatement in the R + BLF group was signiﬁcantly inhibited compared with the R + Veh group. Specifically, postretrieval intra-AI BLF resulted in long-lasting CPP impairment, as the CPP did not re-express in the 14-day post-BLF treatment test or after a priming injection of morphine. BLF disrupted the reconsolidation of CPP rather than it being due to extinction in our study procedure.

Recent findings support the idea that insular responses to drug cues correlate with drug cravings (Perini et al., 2019); in addition to its role in relapse, the insula promotes visceral-emotional functions and decision making (Paulus et al., 2005; Spierling et al., 2019). Furthermore, the IC comprises three distinct cytoarchitectural subregions ordered from the dorsal to ventral cortex, known as the GI, dysgranular (DI), and AI (Purger et al., 2009; Pushparaj et al., 2015), which can be considered a region with extensive homology with the human anterior and posterior insula in terms of connectivity and cytoarchitectonics (Allen et al., 1991; Singer et al., 2009). The AI is the main output domain of the IC, which can send projections to the amygdalo-frontostriatal circuitry (critical in drug addiction) and receive inputs from the polysensory regions of the GI and the adjacent DI (Shi and Cassell, 1998; Gabbott et al., 2003; Reynolds and Zahm, 2005; Kimura et al., 2010; Contreras et al., 2012). From the perspective of nerve fiber connections between these regions, the AI is more likely to participate in behavioral addiction (Moschak et al., 2018). Moreover, it is an indispensable and ideal region that can influence hedonic, motivational, and executive cognitive information processing in relapse circuitry. The functional counterpart is the anatomic fiber projection. Corresponding to the multiple functions involved in drug addiction are its outputs, which are directed at several brain regions including the dorsomedial prefrontal cortex (PFC), lateral orbitofrontal cortex (lOFC), basolateral amygdala (BLA), hippocampus, and nucleus accumbens (NAc) and are closely involved in drug context-induced cocaine-seeking behavior (Naqvi et al., 2014).

There is much evidence suggesting that the consolidated memory can, at least partially, be modified, updated, or eliminated following retrieval and the subsequent reconsolidation processes (Shahveisi et al., 2019). In addition, CPP provides a convenient paradigm for memory reconsolidation research (Reichelt and Lee, 2013). However, there has been no conclusive evidence that the manipulation of memory reconsolidation increases the amount of time spent in the morphine chamber. The possible reason is as follows: the CPP in our study was conducted using a biased protocol. All mice naturally preferred the black chamber over the white chamber. However, following morphine conditioning, the natural aversion to the white chamber and the desire for the drug, leading to preference for the white chamber, reached a dynamic balance. Although the reward memory was partially strengthened after retrieval, it could not break the balance; thus, the amount of time spent in the morphine-conditioned compartment did not increase.

Indeed, previous studies have demonstrated that inactivation of the AI shifts the preference toward options with greater reward frequency and lower punishment in rodents (Ishii et al., 2012; Pushparaj et al., 2015). More importantly, the AI selectively promotes the reconsolidation of drug memories established in Pavlovian models and may be vital for the retrieval of cue/drug-related emotional associations and the anticipation of approaching drug experiences (Liu et al., 2016). Additionally, our findings are consistent with previous reports in which we showed that a single postretrieval microinjection of BLF could disrupt memory reconsolidation in CPP tasks. Importantly, a novel aspect of this study was that BLF has clinical applicability (Brebner et al., 2002), contributing to the treatment of addiction in human trials. Notably, one study found that following reward memory retrieval, a cocktail of GABA agonists (including BLF) induced pharmacological inactivation of the AI but failed to impair subsequent cocaine-memory reconsolidation (Arguello et al., 2017), which seems to be contrary to the conclusion of our study. The following are possible reasons to explain the different results. First, different addictive drugs were selected in the two studies (i.e., morphine versus cocaine). Although cocaine and morphine are both the most commonly used addictive drugs in preclinical studies, they have different metabolic profiles in drug-addicted individuals, which may be attributed to the different actions of the drugs on the brain reward circuitry, the resulting adaptation and behavioral performance (Zaitsu et al., 2014). In addition, different rodent addiction paradigms were used (i.e., CPP paradigm versus cocaine self-administration paradigm). Each paradigm has unique advantages and disadvantages, and we can choose between them according to the purpose of the experiment. The main advantage of the CPP over self-administration models is that CPP reinstatement is measured to reflect the retrieval of a drug-cue memory, including recovery of the approach behavior to the context (Blanco-Gandía et al., 2018). In short, different paradigms and drug treatments may lead to different experimental results.

**Figure 10:**
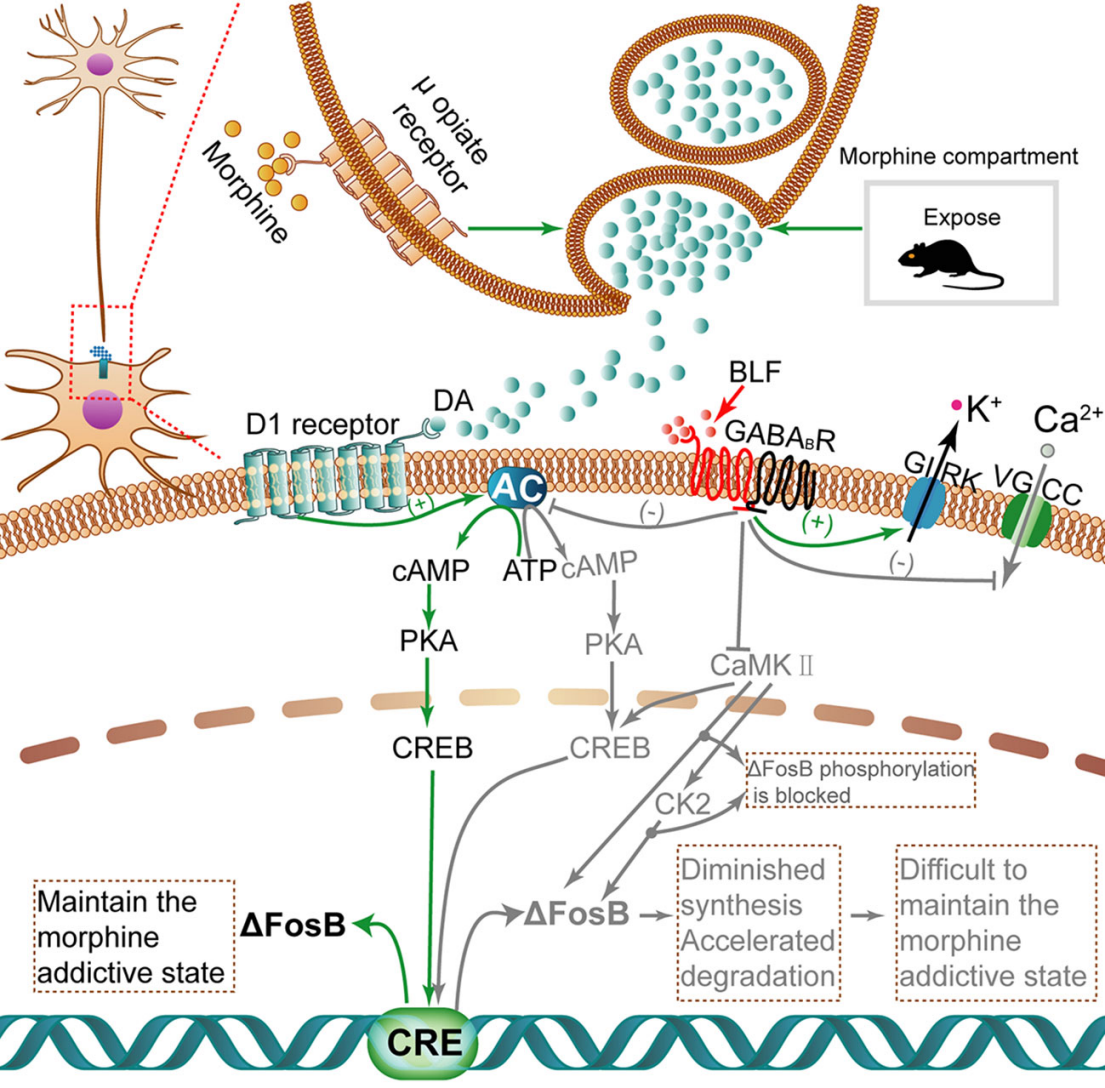
The activation of ΔFosB transcription with morphine induction and inhibition of ΔFosB transcription *via* microinjection of BLF. When morphine is injected, high dopamine concentrations accumulate in the synapse. Dopamine binds to D1 receptors and then activates the cAMP/PKA/CREB pathway. Finally, the transcription activity of ΔFosB is regulated. Once translated, ΔFosB maintains a morphine addictive state by regulating genes involved in synaptic plasticity, leading to morphine dependence and tolerance. Nevertheless, BLF infusion can inhibit the cAMP/PKA/CREB pathway to diminish the synthesis of ΔFosB and negatively mediate CK2 through some unknown pathways; thus, protein ΔFosB phosphorylation is inhibited, thereby promoting its degradation. Ultimately, the level of ΔFosB eventually may be negatively regulated. Gray arrows and text indicate the pathway inhibited by BLF injection. CaMKII, Calcium-calmodulin dependent protein kinase II; CK2, Casein kinase 2.

Recently, numerous transcription factors have been identified to have some effect in drug addiction procedures; significantly, as shown in **Figure 10**, ΔFosB is induced in the brain's reward regions by exposure to almost all psychopharmaceutical drugs and drug-associated cues or contexts (Bourne et al., 2013). Moreover, ΔFosB is a truncated splice variant of FosB; in other words, it lacks the two C-terminal domains of FosB that contribute to rapid protein degradation, which is related to the rapid and transient expression of FosB (Wang et al., 2016). Because ΔFosB is an extremely stable protein, its level per se characterizes a mechanism through which drugs yield long-lasting changes in gene expression at least several weeks after the termination of drug use (Hasbi et al., 2017). Importantly, ΔFosB is a biomarker that can be used to evaluate the efficacy of therapeutic interventions (Ruffle, 2014). Recently, GABA_B_Rs have been identified as potential anti-addictive therapies, and administration of GS39783, a positive allosteric GABA_B_R modulator, inhibited ΔFosB accumulation in mouse brains (Loic et al., 2007). Consistent with the above research, our work suggested that by exploiting the fragility of consolidated memories shortly after being retrieved, the amnesic intervention of BLF injections into the AI shortly after retrieval of the context-drug memory disrupted the drug-cue associations, suggesting that it is a promising approach to the treatment of addiction. Furthermore, these behavioral effects were associated with reduced morphine-associated ΔFosB expression.

As shown in **Figure 10**, ΔFosB transcription can be activated in mice AI after intermittent morphine administration. The activation of dopamine (DA) neurotransmission plays an important role in drug addiction. Above all, repeated drug administration or retrieval of drug-associated contextual memory induced augmentation of dopamine release, which has been implicated in the rewarding properties of drug use (Nakagawa et al., 2011; Fotros et al., 2013). In addition, there is a certain amount of dopamine D1 receptors and GABA_B_Rs in the IC (Coffeen et al., 2010; Wu et al., 2017). Dopamine binds to D1 dopamine G protein-coupled receptors and activates an intracellular G-protein and then activates adenyl cyclase (AC) to produce cAMP (Da Encarnação et al., 2018). cAMP binds to the regulatory domains of protein kinase A(PKA), causing the release of the effector domains (Sathler et al., 2016). The PKA enters the nucleus and phosphorylates CREB. The CREB, a nuclear transcription factor and upstream mediator of ΔFosB induction, binds to the cAMP response element (CRE) (Vialou et al., 2012), regulating the transcription activity of ΔFosB (CARLEZONJR et al., 2005). Once translated, ΔFosB maintains a morphine addictive state by regulating genes involved in synaptic plasticity, leading to morphine dependence and tolerance. The GABA_B_ receptor is a G protein-coupled receptor (GPCR) that mediates slowly and maintains the inhibitory effect (Bettler et al., 2004).

Next, we tried to describe how BLF infusion affects ΔFosB protein levels. First, BLF injection may reduce the protein synthesis of ΔFosB by inhibiting the cAMP/PKA/CREB pathway, the downstream effects of GABA_B_Rs. In contrast with the activation effect of D1R on AC, GABA_B_Rs activated by BLF can inhibit AC. In detail, GABA_B_Rs are broadly expressed in the nervous system and are heterodimeric, formed from two subunits: GABA_B_ receptor 1 and GABA_B_ receptor 2 (Frangaj and Fan, 2018). GABA_B_Rs mediate their inhibitory action by activating inwardly rectifying K+ channels (GIRK), inactivating voltage-gated Ca2+ channels (VGCC) and inhibiting AC (Terunuma, 2018). Specifically, the activation of GABA_B_Rs, such as binding of GABA or BLF, results in the recruitment and activation of Gαi/o proteins. The activated Gαi/o subunits inhibit AC, resulting in lowered cAMP levels, whereas Gβγ subunits activate GIRK channels at postsynaptic sites and inhibit VGCC at presynaptic sites, leading to neuronal inhibition (Chalifoux and Carter, 2011). Finally, the activation of GABA_B_Rs may have an inhibitory effect on the synthesis of a series of key protein factors, including ΔFosB, in their downstream signaling pathway. Second, postretrieval microinjection of BLF may reduce the stability of ΔFosB and promote its degradation by disrupting the process of memory reconsolidation. ΔFosB has a half-life of ∼8 d *in vivo* (Ulery-Reynolds et al., 2009), making it an ideal candidate mechanism for long-term changes in gene expression caused by chronic drug exposure (Eagle et al., 2015). One of the reasons for the unique stability of ΔFosB is that the phosphorylation of ΔFosB at Ser27 protects it from proteasomal degradation (Ulery et al., 2006; Nestler, 2008). The phosphorylation of Ser27 in ΔFosB is regulated by calcium-calmodulin-dependent protein kinase II (CaMKII) and casein kinase 2(CK2) downstream of CaMKII (Ulery et al., 2006; Dai et al., 2009; Robison et al., 2013; Sanz-Clemente et al., 2013). In addition, GABA_B_R activation by BLF inhibits the activity of CaMKII (Kawaguchi and Hirano, 2002). Therefore, the activation of GABA_B_Rs by BLF may directly inhibit the phosphorylation of ΔFosB at Ser27 by inhibiting the activity of CaMKII and possibly indirectly by inhibiting CK2 downstream of CaMKII. Because ΔFosB loses the protective effect of Ser27 phosphorylation, it is more easily degraded by proteasomes. In summary, the microperfusion of BLF may both contribute to inhibiting the synthesis of ΔFosB and accelerating the degradation of already-synthesized ΔFosB, eventually reducing the levels of ΔFosB.

Furthermore, we will try to discuss the following issue: at the end of all behavioral tests (i.e., 15 days after injection of BLF after retrieval), why does only infusion of BLF after morphine reward memory retrieval reduce ΔFosB but not nonretrieval or waiting 6 h after retrieval? According to the theory of memory reconsolidation, the formation of memory is a dynamic process involving several stages. After the initial learning event, the memory goes through a time-related process called consolidation at the protein molecular level. However, once consolidated, memories are considered stable and no longer vulnerable to interference (Ferrara et al., 2019). In contrast, following retrieval, the consolidated memory is labile and unstable (i.e., susceptible to interference) for a limited time, usually less than 6 h (Li et al., 2015; Wright et al., 2020), and requires *de novo* protein synthesis to “restabilize,” a process called reconsolidation that allows the original memory to be modified (Sandrini et al., 2015). In contrast, without retrieval, the memory representation remained in a stable form and was not subject to interference (Liu et al., 2016). It is inferred that infusion of the AI with BLF, activating GABA_B_Rs in the AI, after context-drug memory retrieval immediately contributed to the inhibition of the cAMP/PKA/CREB pathway (downstream of GABA_B_Rs), CaMKII and CK2, phosphorylating hundreds of substrates in the long-term. In contrast, in the nonretrieval or 6 h after retrieval mice, without *de novo*-synthesized protein, the cAMP/PKA/CREB signaling pathway and the expression of CK2 and CaMKII may only be temporarily inhibited. Finally, the levels of ΔFosB in the retrieval mice rather than the nonretrieval or 6 h after retrieval mice were attributable to diminished synthesis and accelerated degradation in the long term.

In conclusion, we provide the first evidence to demonstrate that GABA_B_Rs in the AI play a critical role in the reconsolidation of a CPP. Furthermore, the activation of GABA_B_Rs by intra-AI BLF could eliminate an established CPP by disrupting its reconsolidation, which could keep ΔFosB, a marker of addiction, at a lower level and be used as a novel treatment for weakening associative memories between drugs and drug-related contextual cues and, consequently, reducing the risk of relapse. This work provides new insights into the function of the insula and potential advances for therapeutic intervention.

## Data Availability Statement

All datasets generated for this study are included in the article.

## Ethics Statement

The animal study was reviewed and approved by Animal Research Ethics Committee of Ningxia Medical University.

## Author Contributions

TS and FW designed and supervised this study. KS, QM, HC and CZ carried out main experimental work and prepared the manuscript. YW analyzed the data. SR, SL, DZ, ZH, DW and HY contributed to manuscript revision.

## Funding

This study was supported by the National Natural Science Foundation of China (NSFC, 81660226), the Major Science and Technology Projects of Ningxia (2016BZ07) and the key research project of Ningxia (2018BFG02007).

## Conflict of Interest

The authors declare that the research was conducted in the absence of any commercial or financial relationships that could be construed as a potential conflict of interest.
